# Nuclear N-WASP Induces Actin Polymerization in the Nucleus with Cortactin as an Essential Factor

**DOI:** 10.3390/cells14010059

**Published:** 2025-01-06

**Authors:** Xin Jiang, Purusottam Mohapatra, Maria Rossing, Wenqian Zheng, Olga Zbodakova, Jayashree Vijay Thatte, Claus Storgaard Sørensen, Thu Han Le Phan, Cord Brakebusch

**Affiliations:** 1 Biotech Research and Innovation Center (BRIC), University of Copenhagen, Ole Maaløes Vej5, 2200 Copenhagen, Denmark; xin.jiang@bric.dk (X.J.); purusottam.ucph@gmail.com (P.M.); wqzheng1@outlook.com (W.Z.); olga.zbodakova@ucsf.edu (O.Z.); jayashree.thatte@bric.ku.dk (J.V.T.); claus.storgaard@bric.ku.dk (C.S.S.); thu.lephan@bric.ku.dk (T.H.L.P.); 2 Center for Genomic Medicine, Rigshospitalet, University of Copenhagen, Blegdamsvej 9, 2100 Copenhagen, Denmark; caroline.maria.rossing@regionh.dk; 3 Department of Clinical Medicine, University of Copenhagen, 2200 Copenhagen, Denmark

**Keywords:** nuclear F-actin, N-WASP, cortactin

## Abstract

Nuclear actin polymerization was reported to control different nuclear processes, but its regulation is poorly understood. Here, we show that N-WASP can trigger the formation of nuclear N-WASP/F-actin nodules. While a cancer hotspot mutant of N-WASP lacking the VCA domain (V418fs) had a dominant negative function on nuclear F-actin, an even shorter truncation mutant found in melanoma (R128*) strongly promoted nuclear actin polymerization. Nuclear localization of N-WASP was not regulated by the cell cycle and increasing nuclear F-actin formation by N-WASP had no obvious influence on replication. However, nuclear N-WASP/F-actin nodules colocalized partially with RNA Pol II clusters. N-WASP-dependent actin polymerization promoted the maturation of RNA Pol II clusters, with the short truncation mutant R128* unexpectedly showing the strongest effect. Nuclear N-WASP nodules including V418fs colocalized with WIP and cortactin. Importantly, cortactin binding was essential but not sufficient for F-actin formation, while WIP binding was required for actin polymerization by R128*. These data reveal a cortactin-dependent role for N-WASP in the regulation of nuclear F-actin and indicate contrasting nuclear effects for N-WASP mutants found in cancer.

## 1. Introduction

N-WASP is a ubiquitously expressed multidomain protein able to promote actin polymerization via its C-terminal VCA domain by interaction with Arp2/3 [1]. In the autoinhibited, closed conformation, the VCA domain interacts with the N-terminal region. The binding of Cdc42-GTP to the central CRIB domain and of phosphoinositides to the adjacent basic region opens N-WASP and enables actin polymerization. A WH1 domain at the N-terminus binds to proteins of the WIP family. A lack of N-WASP leads to early embryonic lethality in mice, indicating essential functions during development [2,3]. On a cellular level, N-WASP is involved in endocytosis, vesicle trafficking, and protrusion formation by localized regulation of F-actin formation [1].

N-WASP is found both in the cytoplasm and the nucleus and has been described to regulate transcription and chromatin modification [4,5,6,7]. A closely related molecule, WASP, was reported to trigger F-actin formation following DNA damage, promoting homology-directed repair [8,9,10]. Experiments with nuclear extracts suggested that N-WASP could contribute to nuclear F-actin formation, but more direct evidence is lacking [5]. Using Lifeact-transfected cells to visualize F-actin, N-WASP was suggested to induce nuclear actin filaments downstream of T cell receptor-triggered Ca^2+^ signaling [11]. However, Lifeact expression was shown to strongly promote actin polymerization, which makes data obtained with this tool difficult to interpret [12].

Recent studies have suggested that nuclear F-actin formation is involved in addition to DNA repair in chromatin motility, chromatin architecture, replication stress, transcription, and development, raising interest in the molecular pathways controlling nuclear actin polymerization [13,14,15]. Although actin levels in the nucleus are lower than in the cytoplasm and nuclear F-actin is less readily detectable, the Grosse group proved its existence by elegant experiments involving not only a novel F-actin-detecting probe but also phalloidin staining of fixed cells, which excludes any detection-related alteration of nuclear F-actin that might occur with F-actin binding probes in living cells [16]. The regulation of nuclear F-actin formation, however, is only poorly understood.

As defects in DNA replication and repair are intimately linked with cancer formation, nuclear actin polymerization might also be linked with malignant disease. Interestingly, cancer genome sequencing revealed the existence of two hotspot mutations of N-WASP, which both lack the actin polymerization-inducing VCA domain: V422fs, found in the uterus and gastrointestinal tract cancers, and R131*, found in melanoma and colon cancer [17]. V422fs is always in an open conformation but is unable to directly promote actin polymerization. R131* is a truncation mutant consisting mainly of the N-terminal WH1 domain.

In order to elucidate the role of N-WASP in nuclear F-actin formation, we expressed HA-tagged wild-type and mutant forms of N-WASP in U2OS osteosarcoma cells or in N-WASP–null primary keratinocytes and evaluated the effect on nuclear actin polymerization.

We demonstrate that N-WASP can promote nuclear actin polymerization in an Arp2/3-dependent manner. Colocalizing nodules of N-WASP and F-actin were detected in the nucleus of N-WASP-overexpressing cells, but no fibrillar F-actin. The mouse orthologs of the two VCA-domain-deficient N-WASP mutants enriched in cancer showed contrasting effects with respect to nuclear actin polymerization, suggesting that a potential cancer-promoting effect is not caused by a common alteration of nuclear F-actin. Nuclear N-WASP nodules colocalized with WIP and cortactin, and cortactin was found to be essential for N-WASP-dependent F-actin formation. The comparison of these in vitro data with phenotypes of N-WASP KO and cortactin KO mice provides insight into the potential in vivo function of N-WASP-dependent nuclear actin polymerization.

## 2. Materials and Methods

### 2.1. Mice

Mice with a keratinocyte-restricted deletion of N-WASP were produced as described previously [18]. All mice were kept in an Association for Assessment and Accreditation of Laboratory Animal Care International, an accredited animal house under specific pathogen-free conditions.

### 2.2. Cells

HEK293 and the human osteosarcoma cell line U2OS (ATCC, HTB-96; Manassas, VA, USA) were grown in Dulbecco’s modified Eagle’s medium with 10% fetal bovine serum (Cytiva HyClone, SV30160.03 1% (*v/v*); Cytiva, Freiburg, Germany), as well as Penicillin (100 U) and Streptomycin (100 µg/mL) (Gibco, 15140-122; Thermo Fisher Scientific, Roskilde, Denmark). U2OS cells were kept in a humidified 37 °C incubator with 5% CO_2_. To generate iCas9 U2OS cells, early passage U2OS cells were transduced with lentivirus containing inducible Cas9 and blasticidin resistance gene (Horizon #CAS11229; Horizon Discovery, Lafayette, CO, USA). Lentivirus was added at a multiplicity of infection (MOI) of 2 in the presence of 8 µg/mL polybrene. Following transduction, iCas9 U2OS cells were maintained in growth media supplemented with 5 µg/mL blasticidin for selection. U2OS cells stably expressing GFP–NLS–Nuclear Actin–chromobody were generated by transfecting U2OS cells with 2 µg of plasmid DNA-encoding GFP–NLS–Nuclear Actin–chromobody (#acg-n; Chromotek, Planegg-Martinsried, Germany). Forty-eight hours post-transfection, neomycin selection was initiated and maintained for one week to enrich cells with stable expression of the construct. This process resulted in the stable expression of GFP-tagged nuclear actin within the selected U2OS cell population (U2OS–GFP–nAC).

Primary keratinocyte isolation from 4–10-week-old mice with a keratinocyte-restricted KO of N-WASP and culture were carried out as described earlier [19]. For transfection, keratinocytes were plated on collagen and fibronectin-coated coverslips placed in 24 well plates and transfected 4 d after isolation.

### 2.3. N-WASP Expression Vectors

HA-tagged murine N-WASP in pcDNA3.1 (Invitrogen; Thermo Fisher Scientific, Roskilde, Denmark) was mutated by PCR using the following primers: Y253D (Forward: 5′ TCAAAAGTTATTGATGACTTTATTG 3′; Reverse: 5′ TGTTTCTCTGTCTTTAAGCTG 3′), Y253F (Forward: 5′ TCAAAAGTTATTTTTGACTTTATTG 3′; Reverse: 5′ TGTTTCTCTGTCTTTAAGCTG 3′), L229P (Forward: 5′ TTGATATGTGTGGGATCTCTGAG 3′; Reverse: 5′ AAAGATTCTTTGGTTCTGGATCCA 3′); R128* (Forward: 5′ GCAAAAAAGTTCTCAAAAGCAGTTACAG 3′; Reverse: 5′ TTCTTCTTCATTGGCAAAATTAAGAGC 3′), V418fs (Forward: 5′ CTCAGCTGAAAAAATGGAACAGAATAGCC 3′; Reverse: 5′ CACCCTCTCTAATTTGATCCAAAAGAGC 3′). Products were cloned by PCR, and restriction-digest into pCAG-Cre-IRES2-GFP (Addgene, 26646; Addgene, Watertown, MA, USA), and sequenced with two primers (pCAG-F: GCAACGTGCTGGTTATTGTG, and N-WASP-F: ACAGGAGGTGTAGAAGCTGT). For PCR, the Phusion High-Fidelity PCR Kit was used (Thermo Fisher Scientific, F553L; Thermo Fisher Scientific, Roskilde, Denmark).

For the GFP–nAC–chromobody experiments, we overexpressed L229P N-WASP using a transiently transfected lentiviral expression vector based on pRRLSIN.cPPT.PGK-GFP.WPRE (Addgene plasmid #12252), where the GFP was replaced by L229P N-WASP.

### 2.4. Generation of KO Cell Lines

KO cell lines were generated using CRISPR genome editing. A single-guide RNA (sgRNA)-targeting human N-WASP (gene name: WASL) and WIP (gene name: WIPF1)-coding region was designed (guide sequence for WASL: 5′ TGCAGTTATATGCAGCAGAT3′, WIPF1: 5′ TGCAAACGTCGGGGGCGGCG 3′, respectively) using crispor.tefor.net (accessed on 15 February 2022) and selection for the low frequency of predicted off-target effects. A DNA sequence encoding the sgRNA was introduced into the lentiviral Cas9-expressing vector lentiCRISPRv2 (Addgene, 62988; Addgene, Watertown, MA, USA) as described previously [20]. To produce viral particles, HEK293T cells were transfected with the lentiCRISPRv2-WIP together with pVSVG (Addgene, 8454; Addgene, Watertown, MA, USA) and pPAX-2 (Addgene, 12260; Addgene, Watertown, MA, USA). Twenty-four hours post-transfection, a virus containing supernatant was collected and added to U2OS cells, which were selected after 2 d with 1 μg/mL puromycin (Gibco, A1113803; Thermo Fisher Scientific, Roskilde, Denmark).

U2OS cells with a doxycycline-inducible iCas9, were treated with 1 μg/mL doxycycline (Sigma, D3447; Sigma-Aldrich, Søborg, Denmark) in a culture medium 24 h before transfection. Then, a crRNA targeting human cortactin (5′-CGGCAAATACGGTATCGACA -3′; IDT) was transfected together with tracrRNA (IDT) using Lipofectamine RNAiMAX (Thermo Fisher Scientific, 13778; Thermo Fisher Scientific, Roskilde, Denmark) following the instructions of the manufacturer. Then, 3–4 days after transfection, the cells were analyzed for knockout efficiency and subsequently used for experiments.

### 2.5. Western Blotting

Western blotting was performed following standard protocols. For all experiments, cell confluency was around 70%. For antibodies used, see Supplementary Table S1. Proteins were then visualized with the ChemiDoc MP Imaging System (Bio-Rad, Copenhagen, Denmark). All results were quantified using Image Lab 6.1 Software (Bio-Rad, Copenhagen, Denmark) and normalized to the expression of GAPDH.

### 2.6. Transfections

Transfection of U2OS cells and primary mouse keratinocytes was carried out using the Lipofectamine 3000 kit (Invitrogen, L3000015; Thermo Fisher Scientific, Roskilde, Denmark) following the instructions of the manufacturer on 30–40% confluent cells seeded on coverslips placed in 24 well plates. Treatment for 1 h with either DMSO or 100 μM CK-666 (Abcam, ab141231) was performed one day after transfection. Afterwards, cells were fixed and stained as described below (Section 2.7).

### 2.7. Fluorescent Staining

Forty-eight hours after transfection, cells cultured on coverslips were analyzed by fluorescent staining. Cells were first permeabilized with 0.1% Triton X-100 and then fixed with 4% paraformaldehyde for 15 min at room temperature. Coverslips were blocked for 1 h with 5% BSA in PBS before incubation with primary antibodies or Alexa Fluor 647 phalloidin to stain F-actin (Thermo Fisher, A22287, 1:100 dilution; Thermo Fisher Scientific, Roskilde, Denmark) overnight. After washing three times with PBS, the coverslips were incubated with secondary antibodies for 1 h at room temperature, protected from light. Next, the coverslips were incubated with a 5 μg/mL DAPI (Thermo Fisher, D1306, Thermo Fisher Scientific, Roskilde, Denmark) for 5 min. Lastly, the coverslips were washed intensively and mounted on a glass slide with 5 μL of mounting medium (Agilent Dako, S3023; Agilent, Santa Clara, CA, USA). For primary and secondary antibodies used, see Supplementary Table S2.

### 2.8. Image Analysis

Three-dimensional (3D) Z-stacks with a thickness of 0.37 μm were captured using a Zeiss LSM800 confocal microscope equipped with a 40×/1.3 oil objective (for settings, see Supplementary Table S3). The central plane of the nucleus was identified as the Z-stack slice exhibiting the largest nuclear area, as visualized by DAPI staining with ZEN 3.6 software (Carl Zeiss Microscopy) and later used for quantitative analysis. Movies from Z-stacks were made with ZEN 3.6 software. For indicated experiments, imaging was performed using a widefield upright microscope (Zeiss AxioImager M2, Carl Zeiss Microscopy, Oberkochen, Germany) at 20×, 40×, or 63× and Zeiss ELYRA7 Super Resolution Microscope with 63× oil objective. Filter sets were chosen to avoid bleed-through and lack of bleed-through was confirmed by analysis of single stains.

For quantitative image analysis, the CellProfiler software version 4.2.1 [21] was used. Nuclei were identified by DAPI staining and cell area by phalloidin staining (F-actin) surrounding the nuclei. Cell borders were detected using changes in intensity using the minimal entropy algorithm. Cells touching the border of the image were excluded from the analysis. Transfected cells were recognized based on their HA staining intensity in the cell area. To obtain the specific HA staining intensity, the average HA staining intensity of the nucleus and cytoplasm of the untransfected cells (UTF) within the picture was subtracted from the corresponding values of the transfected cells (TF). The following values were measured: DAPI, F-actin (phalloidin), HA intensity, and nuclear and cell area. For DAPI and phalloidin, the intensities of the TF cells were normalized to the average of the corresponding intensities of the UTF cells in that experiment. The nuclear fraction of N-WASP was calculated by dividing the integrated intensity of HA staining in the nucleus by the integrated intensity of HA staining in the total cell. Nuclear levels of F-actin were calculated by measuring the integrated intensity of phalloidin staining in the nucleus.

### 2.9. Cell Cycle Analysis by EdU Incorporation

U2OS cells were transfected following the previously outlined procedure. To assess DNA replication, the Click-iT EdU Alexa Fluor 647 Imaging Kit protocol (Thermo Scientific, C10340; ThermoFisher, Roskilde, Denmark) was adhered to. Briefly, cells were treated with 10 μM EdU in a culture medium for 30 min before fixation. EdU incorporation was detected using Alexa Fluor 647-azide (Thermo Fisher Scientific, Roskilde, Denmark). Subsequent immunostaining involved the use of an HA antibody, followed by visualization using Alexa Fluor 568. DAPI staining was performed for nuclear identification and high-exposure HA staining to detect the cell area of transfected cells. Cell cycle distribution was analyzed with help of Spotfire software (Version 14.1.0).

### 2.10. Serum Starvation and Stimulation Treatment

The cells were initially cultured in DMEM supplemented with 10% FBS. For serum-induction experiments, these cells were subsequently transitioned and kept in serum-free media overnight. Subsequently, the serum-starved cells were exposed to 20% FBS medium for 30 min before imaging. As a control, cells were cultured under normal growth conditions in DMEM supplemented with 10% FBS. Transfections were conducted one day prior to the overnight serum-free incubation.

### 2.11. Statistical Analysis

Data were evaluated using Graph Pad Prism and presented as mean ± standard deviation. Statistical significance was determined either by the two-tailed Student *t*-test for data with only two groups, or by one-way ANOVA with Tukey’s post hoc test for experiments with multiple groups. Significant differences are indicated by asterisks (not significant (ns): *p* ≥ 0.05; *: *p* < 0.05; **: *p* < 0.01; ***: *p* < 0.001; ****: *p* < 0.0001). The data compared within a graph are indicated by the start and end of a line.

## 3. Results

### 3.1. Nodules of Endogenous Nuclear N-WASP Colocalize with Nuclear F-Actin in U2OS Cells

**Figure 1:**
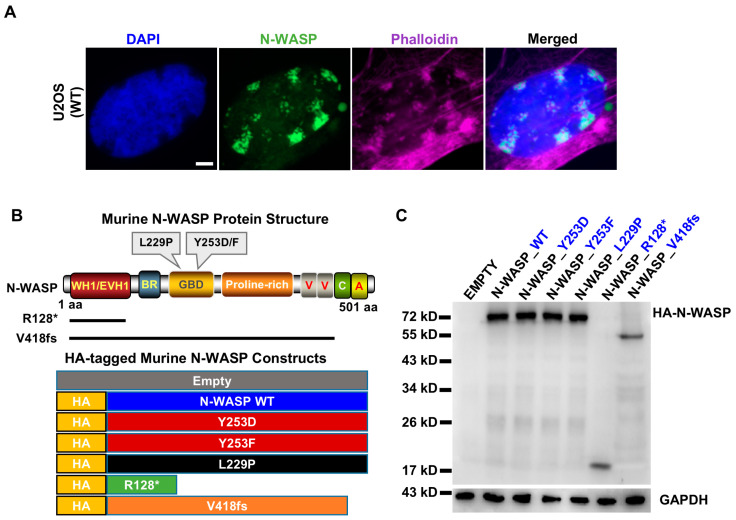
Endogenous nuclear N-WASP colocalizes with F-actin and the establishment of an image-based platform to analyze the role of N-WASP in nuclear actin polymerization. (**A**) Confocal fluorescent staining of untransfected U2OS cells for endogenous N-WASP and F-actin (Scale bar: 5 µm). (**B**) Schematic presentation of murine N-WASP constructs used. N-WASP domains are indicated (WH1/EVH1: WASP homology/Ena-VASP homology domain; BR: Basic region; GBD: GTPase binding domain). All N-WASP constructs had an N-terminal HA tag to facilitate detection. (**C**) Western blot of lysates of U2OS cells transfected with indicated N-WASP constructs or empty vector for HA. GAPDH was used as loading control (n: 1).

To investigate whether endogenous N-WASP has a role in nuclear actin polymerization, we performed immunofluorescent staining and confocal microscopy of U2OS osteosarcoma cells for N-WASP and, using fluorescently labeled phalloidin, for F-actin. While most cells showed diffuse nuclear localization of N-WASP, very few cells (fewer than 1 in 1000) demonstrated one or several nodules of N-WASP apparently located in the nucleus (Figure 1A). These nodules colocacolocalized with F-actin and a decreased DNA density, suggesting that the foci of N-WASP and F-actin are indeed located in the nucleus.

### 3.2. Establishment of a Platform to Assess N-WASP Function in Nuclear F-Actin Formation

To confirm and further investigate the role of N-WASP in nuclear F-actin formation, we generated expression vectors for wild-type murine N-WASP (WT N-WASP) and different mutant forms of it (Figure 1B). Tyrosine 253 was exchanged against phosphorylation-mimicking D (Y253D) or non-phosphorylatable F (Y253F) since it was reported that the tyrosine phosphorylation of N-WASP reduces its nuclear localization [22]. The L229P mutant is preferentially in the open, actin polymerization-promoting conformation [23]. In addition, we tested two truncation mutants that are orthologous to mutations found by genome sequencing in human cancer: R128*, a truncation mutant consisting mainly of the N-terminal WH1 domain (human ortholog R131*; found in melanoma and colon cancer), and V418fs, a mutant carrying a frameshift mutation that results in the loss of the actin polymerization-inducing VCA domain and adds 15 unrelated amino acids (human ortholog V422fs; found in gastrointestinal and uterine cancers). V418fs is, therefore, always in an open conformation but unable to directly promote actin polymerization. For specific detection of the transfected N-WASP molecules, an HA tag was introduced at the N-terminus. All mutant forms of N-WASP were expressed in U2OS cells at the expected molecular weight (Figure 1C).

### 3.3. Nuclear N-WASP Promotes Nuclear F-Actin Formation

**Figure 2:**
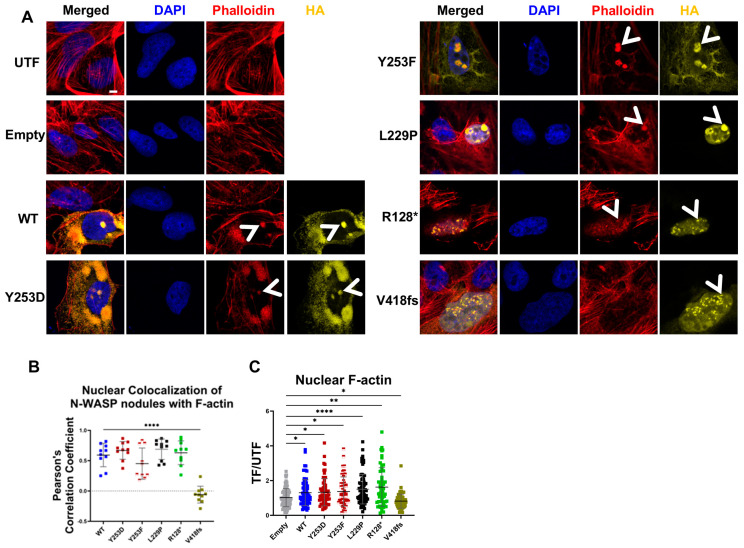
Nuclear N-WASP promotes nuclear F-actin in WT U2OS cells. (**A**) Confocal fluorescent staining of U2OS cells, untransfected (UTF) or transfected with the indicated N-WASP constructs. Cells were stained for DNA (DAPI), F-actin (phalloidin), and transfected N-WASP (HA). Arrowheads indicate nuclear nodules of F-actin or N-WASP (Scale bar: 5 µm). (**B**) Pearson colocalization analysis of nuclear N-WASP nodules with F-actin was performed on ten representative nuclei per group exhibiting clear N-WASP nodules. Each dot represents an individual cell (one-way ANOVA with Tukey’s post hoc test; ****: *p* < 0.0001). (**C**) Normalized nuclear F-actin levels in U2OS cells transfected with the indicated constructs, based on confocal imaging. Each dot represents an individual transfected cell (total number of cells pooled from two independent experiments analyzed for each construct: 88, 73, 60, 51, 86, 58, and 72; one-way ANOVA with Tukey’s post hoc test; *: *p* < 0.05; **: *p* < 0.01; ****: *p* < 0.0001).

To investigate the effect of WT and mutant N-WASP on the formation of N-WASP/F-actin nodules in the nucleus, we performed confocal microscopy of U2OS cells transiently overexpressing N-WASP WT or mutants (Figure 2A). We observed a high cell-to-cell variation in the intracellular localization of the different mutants (Figure S1A). An analysis of the nuclear fraction, which is defined as the fraction of cellular N-WASP (integrated cellular HA intensity) present in the nucleus, indicated a strong decrease in nuclear localization for the tyrosine phosphorylation mimicking the Y253D mutant compared to WT, while the non-phosphorylated Y253F mutant was distributed quite similarly to WT and only showed a trend of increased nuclear localization (Figure S2B). A possible explanation might be that only a small percentage of N-WASP is phosphorylated at Y253 under the conditions tested. Earlier, it was reported that the open conformation of N-WASP exposes a nuclear localization signal that promotes nuclear localization [4,24]. Indeed, both L229P as well as the frameshift mutant V418fs were preferentially, but not exclusively, located in the nucleus (Figure S2B). Unexpectedly, the truncation mutant R128* showed a higher nuclear percentage than wild-type N-WASP, although it lacked the NLS sequence in the basic region [4], possibly because its small size facilitated diffusion to the nucleus. The nuclear percentage of all N-WASP constructs displayed a Gaussian distribution with a single peak.

The overexpression of N-WASP WT, Y253D, Y253F, L229P, R128*, and V418fs resulted in one or multiple nuclear N-WASP nodules of variable size, identified by staining for the HA-tag in 5–10% of the transfected cells (Figure 2A; selected examples of cells with clear nuclear N-WASP nodules indicated by arrowhead). These N-WASP nodules often colocalized with low DAPI staining. We noted that many cells with clear nuclear N-WASP did not show nuclear N-WASP nodules. A visual comparison of N-WASP and F-actin staining suggested a high overlap of nuclear N-WASP nodules with F-actin nodules, with the exception of V418fs. This was confirmed by the mixed color of the nodules in the superimposed images. To quantify the extent of correlation, we applied Pearson’s correlation coefficient. Since this method subtracts the mean intensity from each pixel’s intensity value, it is independent of signal levels and background [25]. To avoid artificially inflated correlations due to the inclusion of regions where both N-WASP and F-actin levels are low and to avoid differences caused by different expression levels in different cells, we restricted the analysis to the areas of nuclear N-WASP nodules identified by CellProfiler in a single cell. All nuclear N-WASP nodules except those of the V418fs mutant strongly colocalized with F-actin as indicated by a high correlation coefficient (Figure 2B). The very low correlation obtained for V418fs supported the validity of the analysis method. This colocalization of N-WASP and F-actin was not caused by bleed-through as shown by control staining (Figure S1C). These data suggest that nuclear N-WASP can induce the formation of nuclear F-actin. Average nuclear F-actin levels were significantly increased for all N-WASP constructs besides V418fs. It was highest for L229 and unexpectedly also R128*, which lacks the Arp2/3-binding VCA domain (Figure 2C). V418fs N-WASP expression resulted in a significant decrease of nuclear F-actin compared to untransfected cells, indicating a dominant negative effect. The dot plot presentation of the data indicated a high cell-to-cell variation of the nuclear F-actin levels for each of the N-WASP constructs (Figure 2C). Of note, no microscopically visible F-actin fibrils were observed and not all cells with strong nuclear localization of N-WASP showed nuclear nodules.

**Figure 3:**
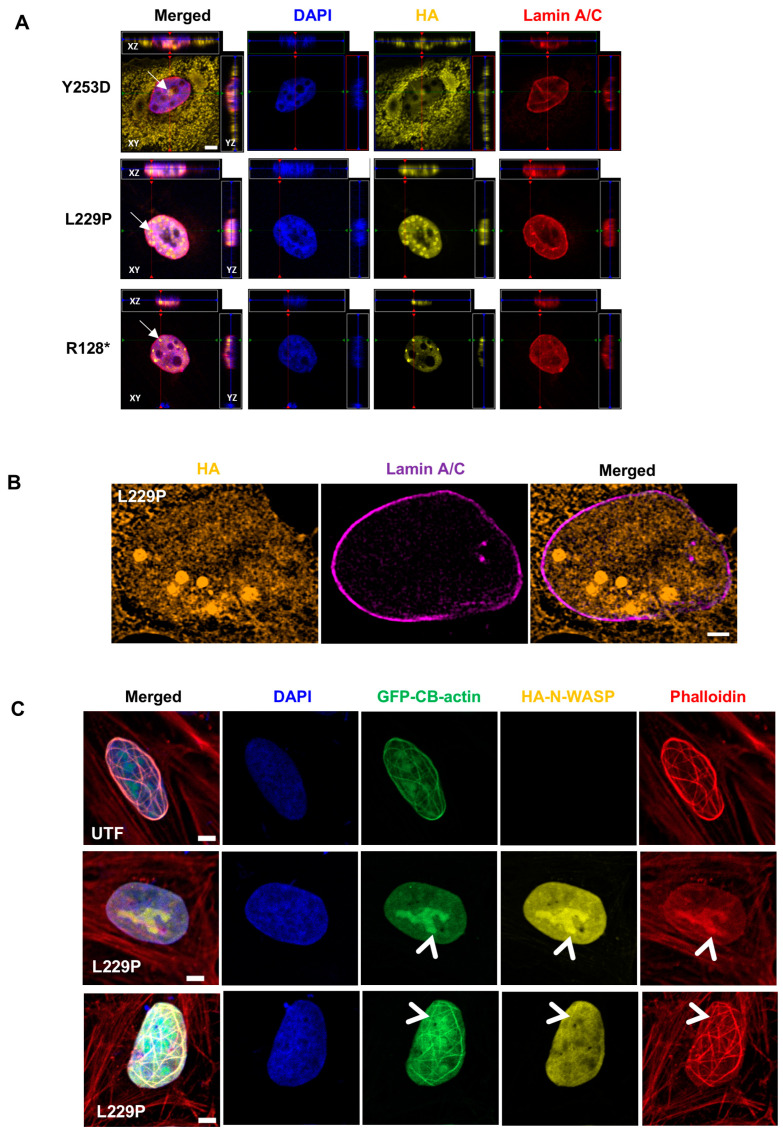
N-WASP promotes nuclear F-actin visualized by actin chromobody. (**A**) Orthogonal views from different planes (x/y, x/z, and y/z) of confocal microscope Z-stacks of U2OS cells transfected with the indicated N-WASP constructs and stained for DNA (DAPI), lamin A/C, and transfected N-WASP (HA). Arrows indicate nuclear N-WASP nodules (scale bar: 5 µm). (**B**) Super-resolution microscopy imaging of L229P-transfected U2OS cells stained for HA (N-WASP) and lamin A/C (Scale bar: 5 μm). (**C**) Representative confocal images of U2OS cells stably expressing GFP–NLS–nuclear actin–chromobody (U2OS–GFP–nAC), untransfected (UTF) or transfected with HA-tagged L229P N-WASP, and stained for DAPI (DNA), GFP + anti GFP (actin), HA (N-WASP), and phalloidin (F-actin). Arrowheads indicate colocalization of HA-tagged N-WASP with nuclear actin structures identified by both actin chromobody and phalloidin staining (scale bar: 5 μm).

To prove that the observed N-WASP/F-actin nodules were indeed located inside the nucleus, we performed several experiments. Firstly, a confocal Z-stack analysis was conducted for N-WASP (HA), F-actin (phalloidin), and DNA (DAPI). Nuclear F-actin nodules mostly overlapped with N-WASP nodules and correlated with nuclear localization as indicated by DAPI staining (Figure S1D; Supplementary Materials Movies). Analyzing the 3D stack in the xy, yz, and xz planes supported that the N-WASP/F-actin nodules were located in the nucleus (Figure S1E). Secondly, a confocal 3D analysis for L229P N-WASP (HA), DNA (DAPI), and the inner nuclear membrane marker lamin A/C confirmed that the nuclear N-WASP/F-actin nodule is located within the nucleus and not in potential grooves or pits of the nuclear membrane (Figure 3A). Thirdly, 2D super-resolution microscopy L229P N-WASP (HA), DNA (DAPI) and lamin A/C confirmed this conclusion (Figure 3B). Finally, nuclear localization of N-WASP/F-actin nodules was assessed by the expression of L229P N-WASP in U2OS cells stably expressing nucleus-targeting GFP–chromobody-staining F-actin. Since fixation weakened GFP fluorescence, we enhanced the chromobody signal by immunofluorescent staining for GFP in the same detection channel (Alexa 488). We noted that cells expressing the chromobodies, but not L229P N-WASP, already showed nodules and fibrils co-staining with phalloidin (Figure 3C; bleed-through control in Figure S1C), which we never observed in phalloidin-stained U2OS cells not expressing F-actin specific chromobodies. This suggests that the chromobodies can promote the formation of nuclear F-actin fibrils. The overexpression of L229P N-WASP increased the percentage of cells with nuclear F-actin nodules or fibrils in chromobody U2OS cells compared to untransfected cells (nodules: from 25% to 35%; fibrils: from 37% to 44%; n: 48/54). Moreover, N-WASP nodules colocalized with nuclear chromobodies against F-actin, proving the nuclear localization of the N-WASP/F-actin nodules (Figure 3C). In addition, L229P N-WASP also colocalized with nuclear F-actin fibrils.

To test whether N-WASP also promotes nuclear actin polymerization in other cell types than U2OS cells, we isolated primary keratinocytes from mice with a keratinocyte-restricted KO of N-WASP, transfected them with the N-WASP constructs, and investigated them by fluorescent staining and quantitative image analysis. As in U2OS cells, we observed in light microscopical analysis increased nuclear F-actin and the formation of nuclear N-WASP nodules colocalizing with F-actin for N-WASP WT, Y253D, Y253F, L229P, and R128*, while for V418fs, no alteration in nuclear F-actin was observed, nor the colocalization of F-actin with V418fs nodules (Figure S2A–C).

These data indicate that N-WASP can induce nuclear F-actin in different cell types, resulting in the formation of nuclear N-WASP/F-actin nodules.

### 3.4. Nuclear N-WASP Forms F-Actin Nodules Through the Arp2/3 Complex

**Figure 4:**
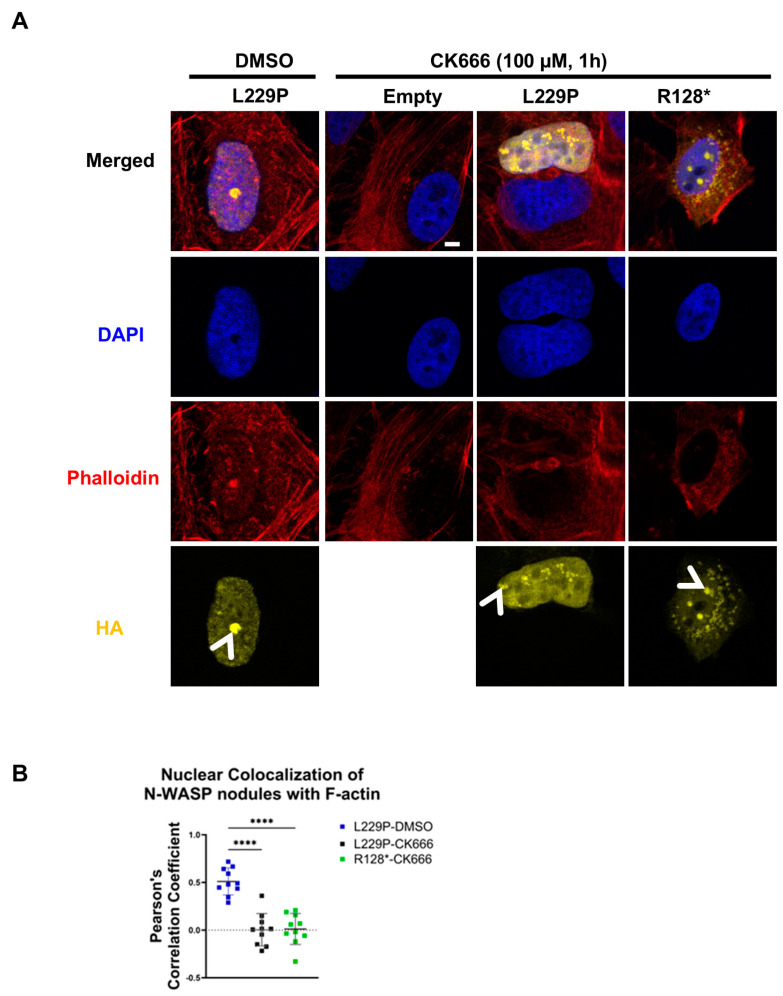
N-WASP promotes nuclear F-actin in an Arp2/3-dependent manner. (**A**) Confocal fluorescent staining of U2OS cells transfected with the indicated N-WASP constructs treated with DMSO or the Arp2/3 inhibitor CK-666 (100 µM, 1 h). Cells were stained for DNA (DAPI), F-actin (phalloidin), and transfected N-WASP (HA). Arrowheads indicate nuclear N-WASP nodules (scale bar: 5 µm). (**B**) Colocalization analysis of nuclear N-WASP nodules with F-actin was performed on ten representative nuclei per group, exhibiting clear N-WASP nodules. Each dot represents an individual cell. Pearson’s correlation coefficient was used to quantify colocalization (one-way ANOVA with Tukey’s post hoc test; ****: *p* < 0.0001).

N-WASP mediates cytoplasmic actin polymerization by association with the Arp2/3 complex. To assess the role of Arp2/3 for the N-WASP-dependent formation of nuclear F-actin nodules, we incubated U2OS cells transfected with L229P or R128* N-WASP for 1 h with 100 μM of the Arp2/3 inhibitor CK-666. This treatment, which reduced nuclear F-actin staining in untransfected cells by 30%, did not prevent the formation of nuclear N-WASP nodules but completely blocked their colocalization with F-actin (Figure 4A,B; Supplementary Materials Movies). These findings show that the constitutively active L229P mutant as well as the VCA-deficient truncation mutant R128*, which cannot directly bind to Arp2/3, promote actin polymerization by activation of Arp2/3.

### 3.5. Nuclear Translocation of N-WASP Is Cell Cycle Independent

**Figure 5:**
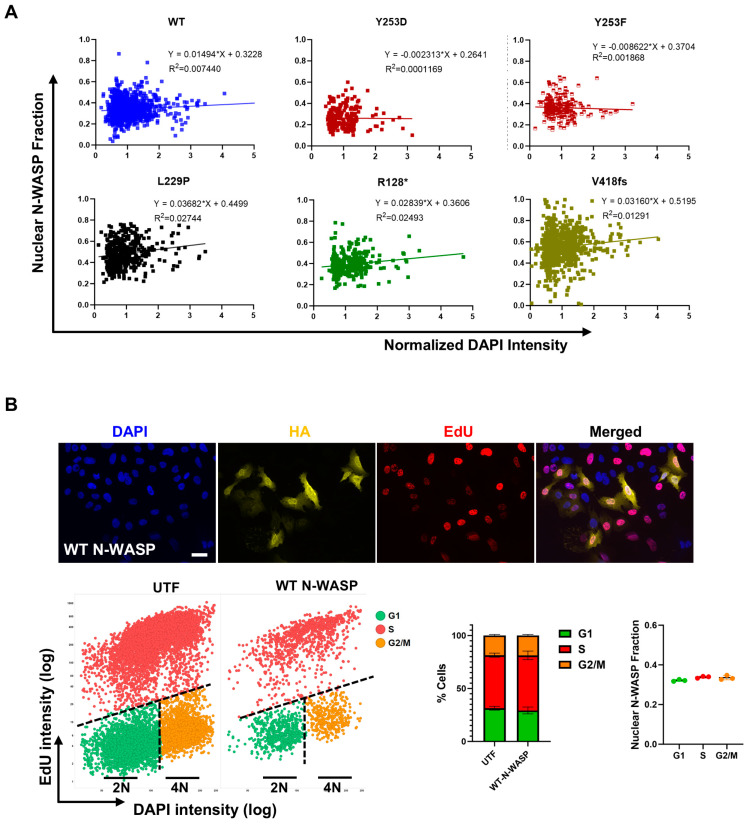
Nuclear N-WASP fraction is independent of cell cycle. (**A**) Correlation of nuclear N-WASP fraction with DNA amount indicated by DAPI intensity. The coefficient of determination, R2, indicates the strength of the linear correlation. (**B**) Cell cycle analysis was performed using an EdU incorporation assay to assess nuclear N-WASP fractions across cell cycle phases. Cells were stained for DAPI (DNA content), EdU (S phase marker), and HA (N-WASP WT). The upper panel shows representative images (scale bar: 20 µm). The scatter plot illustrates gating applied to distinguish G1, S, and G2/M phases presented in the adjacent bar graph. The dot plot on the lower right displays nuclear N-WASP fractions for each cell cycle phase in WT N-WASP-transfected cells (cells analyzed: 12,937, 2041; three independent experiments; one-way ANOVA with Tukey’s post hoc test).

Since nuclear actin polymerization has been described to regulate S-phase progression [26], nuclear growth during early G1 [16], and replication stress response [15], we speculated that the nuclear translocation of N-WASP might be regulated in a cell cycle-dependent manner. However, comparing the nuclear fraction of N-WASP (HA) with DNA amount (DAPI) in single cells, no correlation was found for any of the N-WASP constructs (Figure 5A). Inversely, the overexpression of WT N-WASP did not change cell cycle distribution as determined by an EdU incorporation assay, and the nuclear N-WASP fraction was similar in G1, S, and G2/M (Figure 5B). These data do not indicate a role for N-WASP or N-WASP-dependent nuclear actin polymerization in the cell cycle.

### 3.6. Nuclear N-WASP Is Not Enriched in Nucleoli or Spontaneous DNA Breaks

Nuclear N-WASP/F-actin nodules mostly colocalized with areas of low DNA density, which also characterizes nucleoli. To explore whether nuclear N-WASP nodules might be located in nucleoli, we assessed whether they are colocalizing with the nucleolus marker fibrillarin. To the contrary, we found that overexpressed N-WASP was depleted in nucleoli (Figure S3A).

Nuclear actin polymerization by different nucleation-promoting factors including WASP and N-WASP has been suggested to contribute to the repair of DNA double-strand breaks (DSB) by homologous recombination [27]. Moreover, WASP has been shown to colocalize with DSB breaks [9]. For this reason, we evaluated whether the N-WASP nodules observed in U2OS cells colocalize with spontaneous DSB as detected by γH2Ax staining. However, no colocalization could be observed (Figure S3B), suggesting that the formation of nuclear N-WASP nodules is not related to the repair of DSB.

### 3.7. Nuclear N-WASP Nodules Partially Colocalize with RNA Pol II Clusters

The dominant negative inhibition of N-WASP was reported to prevent the serum-induced formation of RNA Pol II clusters in the nucleus [28]. Generating N-WASP KO U2OS cells (Figure S4A), we show now that the KO of N-WASP decreased the serum-induced increase of number, integrated intensity, and mean intensity of RNA Pol II clusters (Figure S4B,C). Serum treatment, however, still induced a significant increase in the mean intensity even in the absence of N-WASP. These data indicate that N-WASP is important but not essential for RNA Pol II cluster formation.

**Figure 6:**
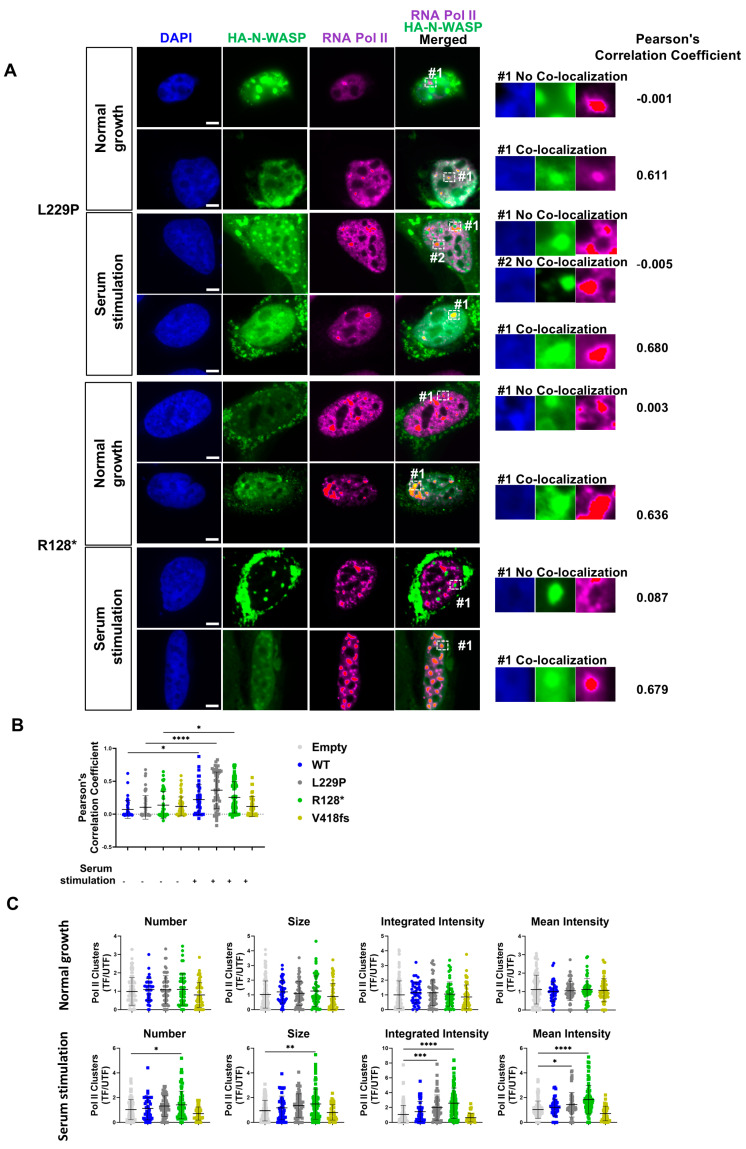
Enhanced RNA Pol II clustering in response to serum stimulation correlates with nuclear actin polymerization of N-WASP. (**A**) Representative images of widefield microscopy of U2OS cells transfected with indicated N-WASP constructs and stained for DNA (DAPI), N-WASP (HA), and RNA Pol II. A white box with a dashed line indicates an RNA Pol II nodule, which is zoomed in on the right. RNA Pol II cluster colocalization with N-WASP nodules shows high cell-to-cell variation. Colocalization occurred in the presence and absence of serum stimulation (scale bar: 5 μm). Colocalization was quantified for these nuclei by Pearson’s correlation coefficient. (**B**) Colocalization of nuclear N-WASP with RNA Pol II for all constructs was determined by Pearson’s correlation coefficient under normal growth (cells analyzed: 141, 47, 61, 52, 62) and serum stimulation conditions (cell analyzed: 120, 46, 56, 77, 40). Data are pooled from three independent experiments and each dot represents a single nucleus (one-way ANOVA with Tukey’s post hoc test; *: *p* < 0.05; **: *p* < 0.01; ***: *p* < 0.001; ****: *p* < 0.0001). (**C**) Quantitative analysis showing normalized number, size, integrated intensity, and mean intensity of RNA Pol II clusters per nucleus in U2OS cells with clear N-WASP nodules under normal growth (cells analyzed: 141, 47, 61, 52, 62) and serum stimulation conditions (cells analyzed: 120, 46, 56, 77, 40). Normalization was performed by dividing transfected (TF) cells by untransfected (UTF) cells.

To test how the formation of nuclear N-WASP nodules is related to RNA Pol II clusters and vice versa, we investigated RNA Pol II cluster formation in U2OS cells transiently overexpressing HA-tagged WT, L229P, R128*, V418fs N-WASP, or an empty control vector. Already in the absence of serum stimulation, we observed high values of Pearson’s correlation coefficient for colocalization of N-WASP nodules with RNA Pol II clusters in some of the N-WASP nodules containing nuclei (Figure 6A, Figure S5). Within a single nucleus, we observed the colocalization with RNA Pol II either for most or for none of the nuclear N-WASP nodules, which was also reflected in Pearson’s correlation coefficients. Serum stimulation increased the fraction of cells with a high Pearson’s correlation coefficient (Figure 6B). This colocalization of nuclear nodules of N-WASP is able to promote F-actin formation with RNA Pol II clusters, suggesting that some RNA Pol II clusters might be able to trigger nuclear N-WASP nodule formation.

Restricting the analysis to cells showing clear nuclear N-WASP nodule formation, we observed a significant increase of RNA Pol II clusters in cells with nuclear nodules of R128* and L229P (Figure 6C). V418fs expression showed a trend of reduced clustering. These data reveal that an increase in N-WASP expression and its actin-polymerizing activity promotes RNA Pol II cluster formation. Since the R128* truncation mutant showed the strongest effect, there is no essential requirement for any N-WASP domain except WH1.

### 3.8. Nuclear N-WASP Nodules CoLocalize with WIP and Cortactin

**Figure 7:**
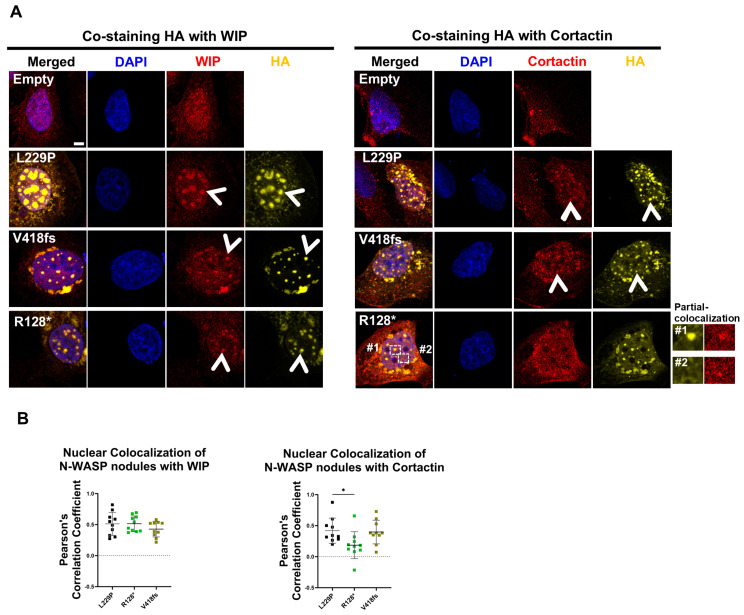
Colocalization of Nuclear N-WASP with WIP and cortactin. (**A**) Confocal images of U2OS cells transfected with indicated N-WASP constructs, stained for DNA (DAPI), N-WASP (HA), and either WIP or cortactin. Arrowheads indicate nuclear N-WASP nodules (scale bar: 5 µm). (**B**) Colocalization analysis of nuclear N-WASP nodules with nuclear WIP and cortactin was performed on ten representative nuclei per group exhibiting clear N-WASP nodules, determined by Pearson’s correlation coefficient. Each dot represents an individual cell (one-way ANOVA with Tukey’s post hoc test; *: *p* < 0.05).

N-WASP can bind via the WH1 domain to WIP. WIP, on the other hand, can directly interact with actin and cortactin and was shown to regulate F-actin levels [29]. Cortactin is an actin polymerization-promoting factor able to bind actin, N-WASP, and WIP, but also many other proteins [30]. To assess whether the N-WASP/F-actin nodules colocalize with WIP and cortactin, we transfected U2OS cells with N-WASP L229P, R128*, and V418fs and stained for DNA (DAPI), transfected N-WASP (HA), and WIP or cortactin. Confocal microscopy and Pearson correlation revealed that nuclear nodules of L229P always colocalized with WIP and cortactin, while R128* colocalized with WIP, but to a significantly lesser extent with cortactin (Figure 7A,B). Interestingly, nuclear V418fs nodules colocalized with both WIP and cortactin, although they did not colocalize with F-actin (cf. Figure 2A,B). These data suggest that nuclear WIP and cortactin might be involved in the formation of nuclear N-WASP/F-actin nodules.

### 3.9. Nuclear Actin Polymerization by R128* N-WASP Is Dependent on WIP

**Figure 8:**
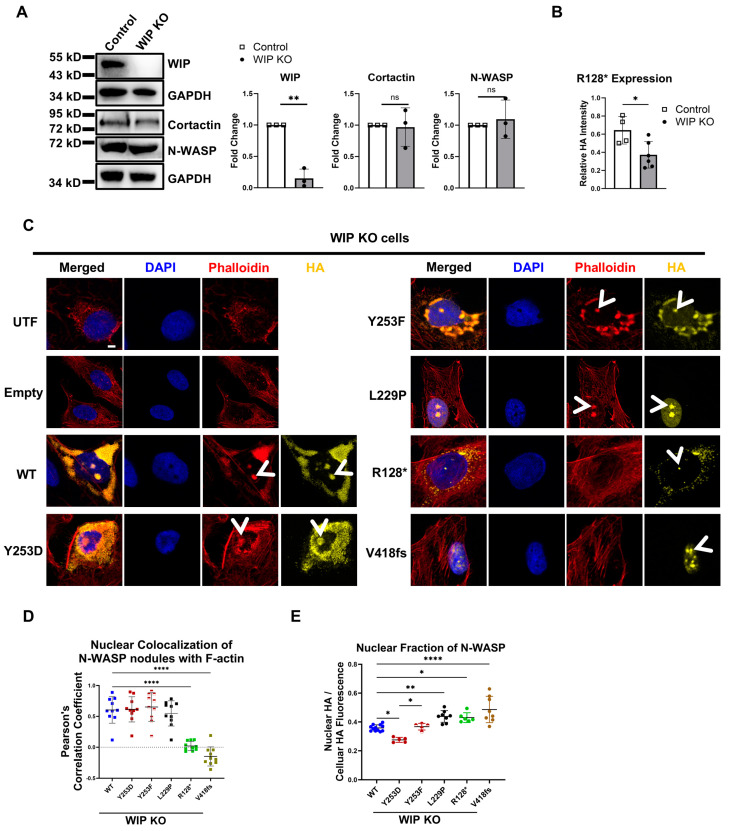
WIP is not essential for N-WASP-dependent nuclear actin polymerization. (**A**) Western blot for indicated proteins showing efficient loss of WIP protein in WIP KO U2OS cells (*n* = 3; two-tailed Student’s *t*-test; ns: *p* > 0.05; **: *p* < 0.01). (**B**) R128* expression analysis in WIP KO relative to parallel transfected WT cells analyzed by quantification of HA intensity of transfected cells (n: 4/6; two-tailed Student’s *t*-test; *: *p* < 0.05). (**C**) Confocal fluorescent microscopy of WIP KO U2OS cells, untransfected (UTF) or transfected with the indicated N-WASP constructs and stained for DNA (DAPI), F-actin (phalloidin), and transfected N-WASP (HA). Arrowheads indicate nuclear N-WASP or F-actin nodules (scale bar: 5 µm). (**D**) Colocalization analysis of nuclear N-WASP nodules with nuclear F-actin in WIP KO cells was performed on ten representative nuclei per group exhibiting clear N-WASP nodules, determined by Pearson’s correlation coefficient. Each dot represents an individual cell (one-way ANOVA with Tukey’s post hoc test; ****: *p* < 0.0001). (**E**) Nuclear N-WASP fraction based on fluorescence staining in WIP KO U2OS cells transfected with the indicated constructs, analyzed via wide-field microscopy (*n* = 12, 5, 5, 8, 6, 8; total cells analyzed: 1060, 849, 1053, 478, 360, 467. One-way ANOVA with Tukey’s post hoc test *: *p* < 0.05; **: *p* < 0.01; ****: *p* < 0.0001).

To understand whether WIP contributes to nuclear actin polymerization by N-WASP, we generated U2OS cells lacking WIP by CRISPR-mediated gene KO. Western blot analysis indicated efficient KO of WIP in a polyclonal cell mixture (Figure 8A). Protein amounts of endogenous N-WASP or cortactin were not affected by the KO of WIP, and nuclear F-actin levels were unchanged. Interestingly, a quantified analysis of the HA fluorescence in the transfected cells suggested a decreased protein amount of R128* in the absence of WIP (Figure 8B). Even in the absence of WIP, nuclear N-WASP nodules were found for all N-WASP constructs (Figure 8C; Supplementary Materials Movies). Nuclear nodules of V418fs and R128*, however, showed no colocalization with F-actin, in contrast to N-WASP WT, Y253D, Y253F, and L229P (Figure 8D). An analysis of widefield microscopy images confirmed that WIP is not required for the nuclear localization of N-WASP (Figure 8E).

These data indicate that WIP is required for R128*-dependent nuclear actin polymerization, but not for the F-actin formation by VCA domain-containing forms of N-WASP.

### 3.10. Nuclear Actin Polymerization by N-WASP Function Is Dependent on Cortactin

**Figure 9:**
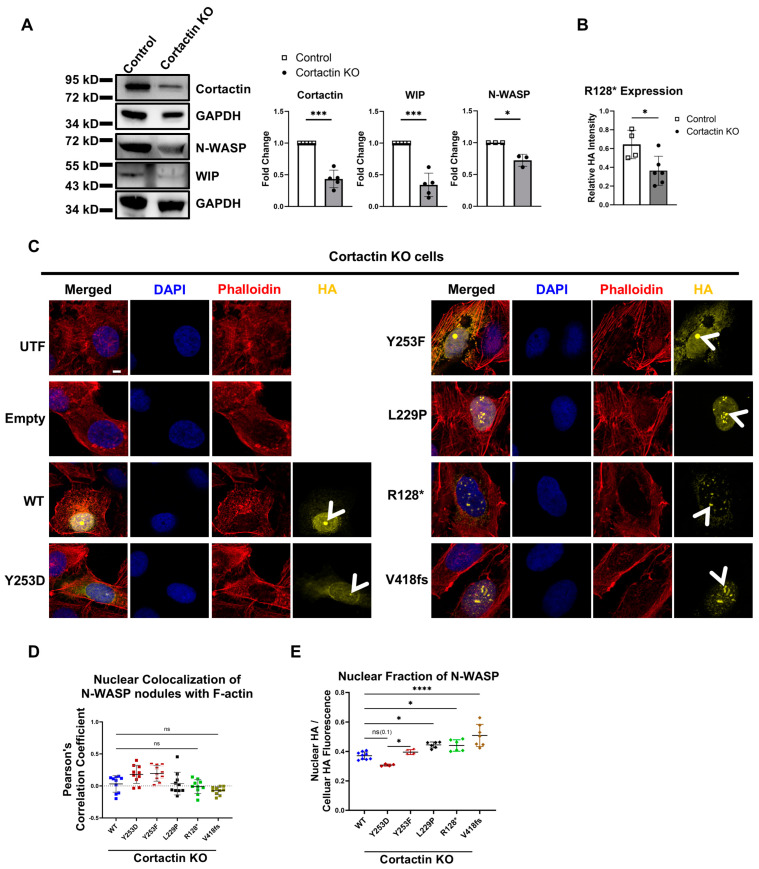
Cortactin is crucial for N-WASP-dependent nuclear actin polymerization. (**A**) Western blot for indicated proteins showing efficient loss of cortactin protein in cortactin KO U2OS cells (n: 3/5; two-tailed Student’s *t*-test, *: *p* < 0.05; ***: *p* < 0.001). (**B**) R128* expression analysis in cortactin KO relative to parallel transfected WT cells analyzed by quantification of HA intensity of transfected cells (n: 4/6; two-tailed Student’s *t*-test; *: *p* < 0.05). (**C**) Confocal fluorescent microscopy of cortactin KO U2OS cells, untransfected (UTF) or transfected with the indicated N-WASP constructs and stained for DNA (DAPI), F-actin (phalloidin), and transfected N-WASP (HA). Arrowheads indicate nuclear N-WASP nodules (scale bar: 5 µm). (**D**) Colocalization analysis of nuclear N-WASP nodules with nuclear F-actin in cortactin KO cells was performed on ten representative nuclei per group exhibiting clear N-WASP nodules, determined by Pearson’s correlation coefficient. Each dot represents an individual cell (one-way ANOVA with Tukey’s post hoc test; not significant (ns): *p* > 0.05). (**E**) Nuclear N-WASP fraction based on fluorescence staining in cortactin KO U2OS cells transfected with the indicated constructs, analyzed via wide-field microscopy. Each dot represents data from an independent experiment, with over 40 cells analyzed per experiment (*n* = 9, 4, 4, 6, 6, 7; total cells analyzed: 970, 757, 801, 886, 248, 321; one-way ANOVA with Tukey’s post hoc test; not significant (ns): *p* > 0.05; *: *p* < 0.05; ****: *p* < 0.0001).

To test the involvement of cortactin in N-WASP-mediated nuclear actin polymerization, we generated polyclonal cortactin-deficient U2OS cells using an inducible CRISPR-Cas9. Western blot indicated the efficient deletion of the cortactin gene in most of the cells (Figure 9A). The loss of cortactin resulted in a strong reduction of WIP protein (Figure 9A). Also, the endogenous N-WASP protein was slightly decreased in the absence of cortactin. R128* protein levels in transfected cortactin KO cells were decreased compared to WT cells, similar to the observation in WIP KO cells and in line with the reduction of the WIP protein in the absence of cortactin (Figure 9B).

In the absence of cortactin, nuclear N-WASP nodules were detected for WT, Y253D, Y253F, L229P, R128*, and V418fs (Figure 9C; Supplementary Materials Movies). However, we could not detect a significant colocalization of F-actin in any of these mutants, suggesting that cortactin is essential for nuclear actin polymerization by N-WASP (Figure 9D). Cortactin KO did not affect the nuclear localization of the N-WASP constructs, as determined by widefield microscopy (Figure 9E).

These data identify cortactin as an essential co-factor for nuclear F-actin formation by N-WASP.

## 4. Discussion

In this study, we show that nuclear N-WASP nodules highly overlap with F-actin nodules, suggesting that nuclear N-WASP can contribute to nuclear actin polymerization. Pearson correlation coefficients for nuclear N-WASP nodules with F-actin were similar for N-WASP WT, Y253D, Y253F, and L229P, suggesting that these N-WASP forms are, to a similar extent, in an open, actin polymerization-promoting conformation when present in nodules. Earlier, it was reported that the open conformation of N-WASP might expose a potential cortactin binding site at the proline-rich region [31]. Indeed, nuclear nodules of the open conformation mutant V418fs colocalized with cortactin, although this mutant could not polymerize F-actin. An N-WASP mutant lacking the potential cortactin binding site (R128*) showed a significantly lower colocalization with cortactin.

Nuclear N-WASP could form nodules independent of actin polymerization, cortactin, WIP, and the VCA domain of N-WASP. However, in cells with a high nuclear expression of constitutively active L229P N-WASP, these nodules were observed only in a fraction of cells, indicating that the nuclear localization of open conformation N-WASP is not sufficient for nodule formation, which indicates other rate-limiting regulatory pathways. The location of nuclear N-WASP nodules partially overlapped with RNA Pol II clusters under serum stimulation, but not with nucleoli or DSB. Nuclear nodules formed by the polymerization-deficient V418fs mutant, however, did not overlap with RNA Pol II clusters. We did not test whether N-WASP nodules colocalize with Cajal bodies, speckles, or histone marks, which have been shown to be regulated by N-WASP.

L229P N-WASP showed colocalization with nuclear F-actin fibrils detected by chromobodies and fluorescently labeled phalloidin. However, these structures were only found in nuclear chromobody-transfected cells and never in untransfected cells. In contrast, phalloidin-stained nuclear N-WASP/F-actin nodules could be observed in the presence and in the absence of nuclear F-actin chromobodies, indicating that they are not reliant on the presence of chromobodies. The chromobodies, therefore, confirm the formation of nuclear F-actin nodules by N-WASP and suggest a potential for N-WASP contribution to fibrillar nuclear F-actin, at least in the presence of chromobodies. Earlier, N-WASP-dependent, nuclear actin filaments were reported in Jurkat cells transfected with the Lifeact probe, which later was found to promote nuclear F-actin formation by competing with actin-binding proteins such as cofilin or myosin [11,12,32]. Our data indicate that F-actin chromobodies may also promote nuclear F-actin formation. These data show that while nuclear actin probes expressed in living cells are excellent in distinguishing nuclear from cytoplasmic F-actin, the results obtained need to be carefully compared to the staining of fixed cells by fluorescently labeled phalloidin to avoid misinterpretations.

Unexpectedly, the truncation mutant R128*, which lacks an NLS-sequence and VCA domain, was able to induce nuclear N-WASP/F-actin nodules similar to L229P. As this also occurred in N-WASP KO keratinocytes, this effect was not dependent on interaction with endogenous wild-type N-WASP. Nuclear R128* nodules showed a similar colocalization with WIP as the other N-WASP constructs but a reduced colocalization with cortactin. Colocalization with F-actin, however, was similar to the other N-WASP constructs with an Arp2/3 binding VCA domain and dependence on Arp2/3, as shown by inhibitor experiments. In contrast to the other N-WASP constructs, F-actin formation by R128* was entirely dependent on WIP. The binding of WIP to N-WASP was reported to stabilize the closed conformation of N-WASP and to inhibit F-actin formation [33]. On the other hand, WIP can bind to actin, profilin, and cortactin and promote F-actin formation by different mechanisms [27]. In U2OS cells, WIP protein levels were strongly dependent on cortactin expression. As WIP and cortactin can interact, it is possible that WIP is stabilized by cortactin. These data suggest that R128* triggers nuclear F-actin formation in a WIP/cortactin/ARP2/3-dependent manner different from WT N-WASP, although the molecular details need to be determined.

N-WASP V418fs contains the WH1 domain and is present in an open conformation, but in contrast to R128*, it is unable to promote actin polymerization, suggesting that amino acid sequences between the WH1 and the VCA domain inhibit the actin polymerization function of the WH1 domain. The human orthologs of R131* and V422fs are both hotspot mutations in cancer, but with respect to nuclear actin polymerization, they have quite opposite effects, as our results indicate. While R128* promotes nuclear F-actin formation, V418fs exerts even a dominant negative effect on wild-type N-WASP with respect to actin polymerization. Future experiments should test the tumor-promoting function of these N-WASP mutants and whether they are related to their effect on nuclear actin polymerization.

Cortactin was found to be essential in the efficient induction of nuclear actin polymerization by N-WASP but not sufficient, as V418fs colocalized with cortactin but not with F-actin. Previously, it was shown that cortactin promotes cytoplasmic actin polymerization by N-WASP, although the mechanism is debated. Other than the direct binding of cortactin to N-WASP [34,35,36], an indirect mechanism was suggested, where N-WASP has first to detach from F-actin before cortactin can bind to F-actin and further promote actin polymerization [37]. The reduced N-WASP protein levels in cortactin-deficient U2OS cells might indicate a stabilizing complex formation between N-WASP and cortactin. Interestingly, the nuclear localization of cortactin can be controlled by acetylation [38]. This suggests a pathway for regulating N-WASP-dependent nuclear F-actin formation via controlling cortactin localization. Cortactin might also promote actin polymerization in an N-WASP-independent manner [39]. Many proteins have been described to bind to cortactin, resulting in a complex regulatory network [30].

What do these data tell us about the in vivo function of N-WASP-dependent actin polymerization in the nucleus? It is difficult to directly address this question since it is technically challenging to specifically interfere with N-WASP-dependent actin polymerization in the nucleus in vivo. However, our finding that nuclear F-actin formation by N-WASP is crucially dependent on cortactin allows us to compare N-WASP and cortactin KO phenotypes in mice to obtain some understanding. N-WASP KO in mice leads to embryonic lethality [2,3]. In addition, the cell-type-specific KO of N-WASP resulted in multiple phenotypes, including in keratinocytes in hair loss, chronic inflammation, loss of keratinocyte stem cells, and increased senescence [6,7,18]. Of four cortactin KO mouse strains made, three were viable and fertile and did not show any reported skin phenotype [40,41,42] (www.mousephenotype.org/data/genes/MGI:99695). These findings suggest that N-WASP-dependent nuclear actin polymerization does not significantly contribute to the developmental or skin phenotypes observed in N-WASP KO mice. Notably, cortactin KO mice displayed impaired endothelial functions [39], which raises the possibility that N-WASP-dependent nuclear actin polymerization could play a role in endothelial cells. Since the frequency of nuclear N-WASP nodules in untransfected cells in standard growth culture was very low, more research is needed to identify conditions where it is increased. Obvious candidates would be diseases where N-WASP expression is highly increased, the N-WASP gene is mutated or amplified, or where the physiological regulation of nuclear actin polymerization is altered. The increased expression of N-WASP has been reported for various cancers and also for Alzheimer’s disease and epilepsy [43,44,45]. In Buruli ulcers, Mycobacterium ulcerans strongly increases N-WASP actin polymerization activity [46]. Based on our data, we would expect an increased frequency of nuclear N-WASP/F-actin nodules under these pathological conditions, which might affect disease progression.

## Data Availability

All data are contained within the manuscript.

## Supplementary Materials

The following supporting information can be downloaded at https://www.mdpi.com/article/10.3390/cells14010059/s1, Figure S1: Confirmation of N-WASP and F-actin nodules within the nucleus; Figure S2: Nuclear N-WASP promotes nuclear F-actin in primary mouse keratinocytes; Figure S3: Nuclear N-WASP nodules do not colocalize with nucleoli or spontaneous γH2AX foci; Figure S4: Partial requirement of endogenous N-WASP in serum-induced enhancement of RNA Pol II clusters; Figure S5. Co-localization of N-WASP nodules with RNA Pol II shows a widespread pattern both in normal growth and serum stimulation; Figure S6. Co-localization of N-WASP L229 nodules with RNA Pol II shows a widespread pattern in serum stimulation; Table S1. List of antibodies used for Western blot; Table S2. List of antibodies used for immunfluorescence; Table S3. Confocal microscopy settings for using the Plan-Apochromat, 40×/ 1.3 oil objective of the Zeiss LSM 800; Figure 2A Z-stack movie; Figure 4A Z-stack movie; Figure 8C Z-stack movie; Figure 9C Z-stack movie; Figure S1D Z-stack movie; Figure S6 Z-stack movie.
