# Supplementary material for: Nuclear N-WASP Induces Actin Polymerization in the Nucleus with Cortactin as an Essential Factor
Source: Cells. 2025 Jan 6;14(1):59. doi: 10.3390/cells14010059 (PMC11720165; doi:10.3390/cells14010059)
Supplement: Supplementary file 1 [file cells-14-00059-s001.zip › Supplementary Material Xin/ Suppl Materials Cells.pdf]

Supplementary Materials

Title:

Nuclear N-WASP induces actin polymerization in the nucleus with cortactin as an essential factor

Authors:

Xin Jiang, Purusottam Mohapatra, Maria Rossing, Wenqian Zheng, Olga Zbodakova, Jayashree Vijay Thatte, Claus Storgaard Sørensen, Thu Han Le Phan, Cord Brakebusch

List of material included:

Supplementary Figures S1-S6

Supplementary Table S1-S3

Supplementary Movies

Figure S1

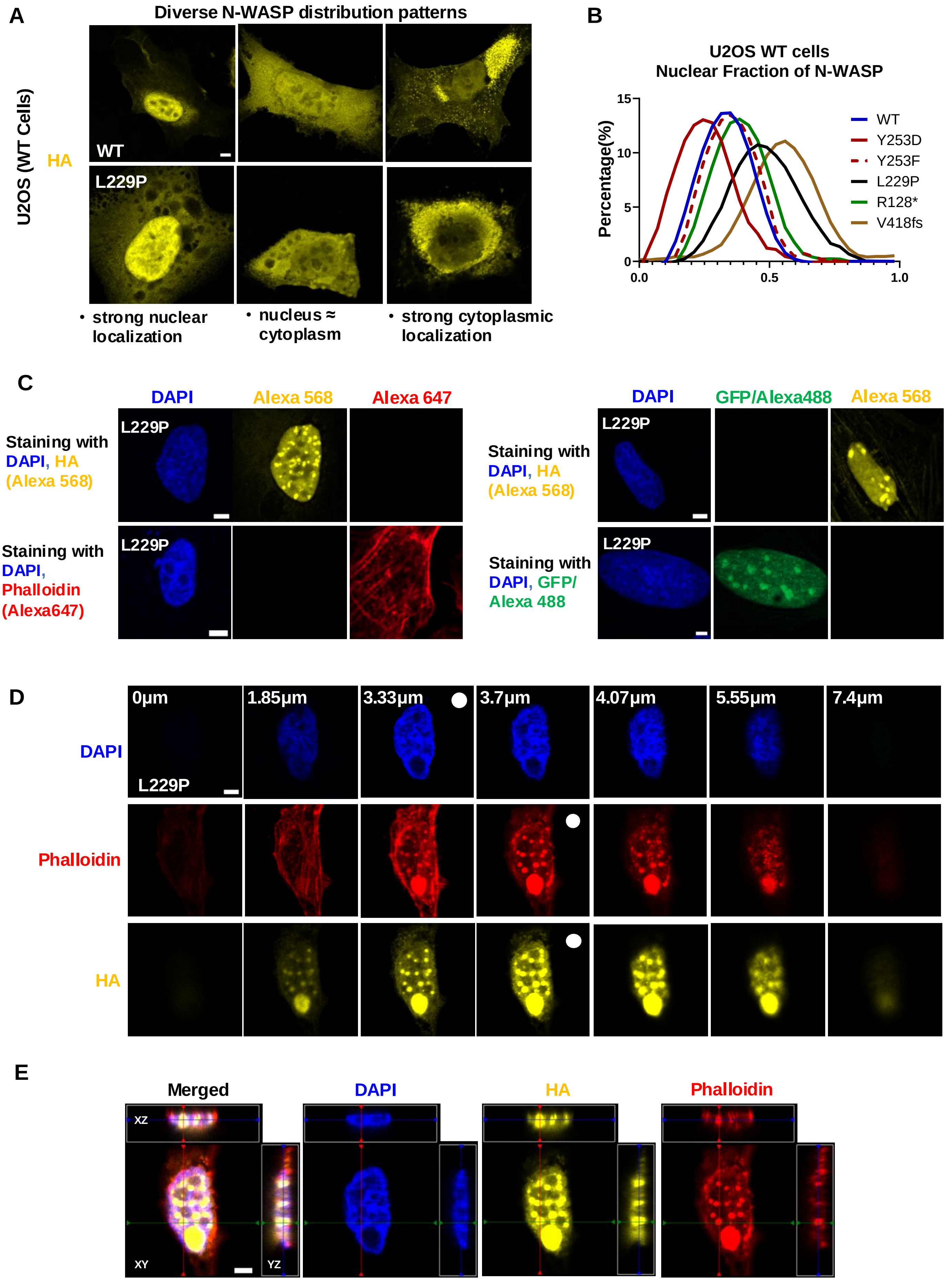
A
Diverse N-WASP distribution patterns
U2OS (WT Cells)
HA
WT
L229P
• strong nuclear localization
• nucleus ≈ cytoplasm
• strong cytoplasmic localization
B
U2OS WT cells
Nuclear Fraction of N-WASP
Percentage(%)
15
10
5
0
0.0
0.5
1.0
WT
Y253D
Y253F
L229P
R128*
V418fs
C
DAPI
Alexa 568
Alexa 647
Staining with DAPI, HA (Alexa 568)
Staining with DAPI, Phalloidin (Alexa647)
L229P
DAPI
GFP/Alexa488
Alexa 568
Staining with DAPI, HA (Alexa 568)
Staining with DAPI, GFP/ Alexa 488
D
0µm
1.85µm
3.33µm
3.7µm
4.07µm
5.55µm
7.4µm
DAPI
Phalloidin
HA
L229P
E
Merged
DAPI
HA
Phalloidin
XZ
XY
YZ

Figure S1: Confirmation of N-WASP and F-actin nodules within the nucleus.
(A) High cell-to-cell variation of intracellular N-WASP distribution among U2OS cells transfected with WT and L229P mutant. Confocal fluorescence images show WT U2OS cells stained for transfected N-WASP (HA). (Scale bar 5 μm).
(B) Histogram of nuclear fraction of indicated N-WASP constructs expressed in U2OS cells obtained by quantitative analyses of widefield images by CellProfiler. (n: 217-1168). (C) Validation of no bleed-through between GFP/Alexa 488, N-WASP (HA, Alexa 568) and F-actin (phalloidin, Alexa 647) channels. The upper panel shows confocal fluorescence imaging of U2OS cells transfected with L229P N-WASP, stained for DNA (DAPI) and either N-WASP (HA, Alexa 568) or F-actin (phalloidin, Alexa 647). The lower panel shows U2OS-GFP-nAC cells transfected with L229P N-WASP, stained for DNA (DAPI), and either GFP (actin chromobody, Alexa 488) or N-WASP (HA, Alexa 568) (Scale bar: 5 μm). (D) Z-stack series of confocal pictures of L229P transfected U2OS cells stained for DNA (DAPI), F-actin (phalloidin) and transfected N-WASP (HA) at the indicated z-distances. White circles indicate layers with maximal intensity (Scale bar 5 μm). (E) z-stack of Fig. S1C viewed from different orthogonal planes (x/y, x/z, and y/z). (Scale bar 5 mm).

Figure S2

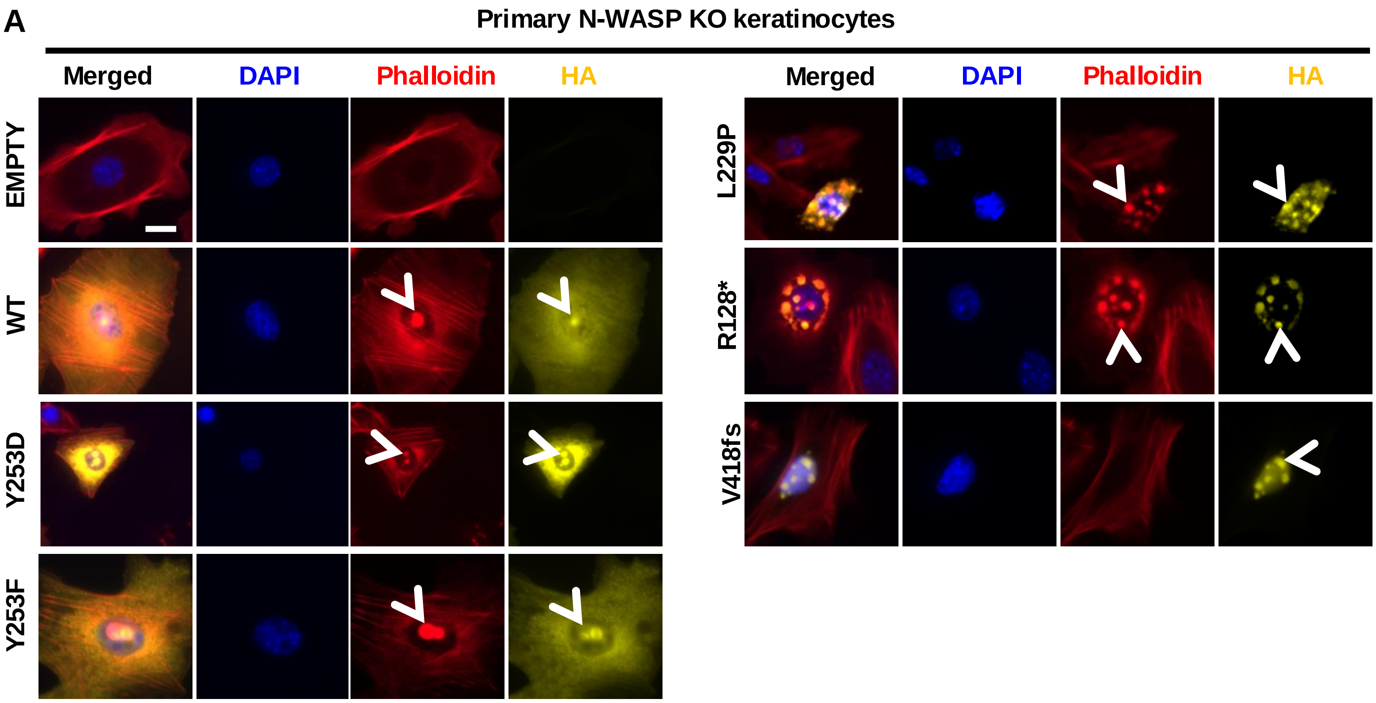

A
Primary N-WASP KO keratinocytes
Merged
DAPI
Phalloidin
HA
EMPTY
WT
Y253D
Y253F
L229P
R128*
V418fs

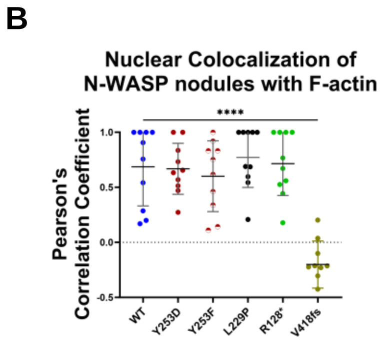

B
Nuclear Colocalization of
N-WASP nodules with F-actin
Pearson's
Correlation Coefficient
****
1.0
0.5
0.0
-0.5
WT
Y253D
Y253F
L229P
R128*
V418fs

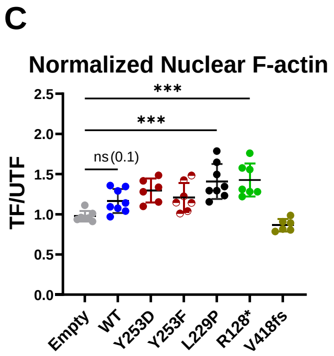

C
Normalized Nuclear F-actin
TF/UTF
***
***
ns (0.1)
2.5
2.0
1.5
1.0
0.5
0.0
Empty
WT
Y253D
Y253F
L229P
R128*
V418fs

Figure S2: Nuclear N-WASP promotes nuclear F-actin in primary mouse keratinocytes.

(A) Wide-field microscopy of fluorescent staining of N-WASP KO primary mouse keratinocytes transfected with the indicated N-WASP constructs. The cells were stained for DNA (DAPI), F-actin (phalloidin), and transfected N-WASP (HA). Arrowheads indicate nuclear N-WASP or F-actin nodules (Scale bar: 20 μm). (B) Ten representative nuclei per group from with clear HA nodules were selected to assess co-localization of nuclear N-WASP with nuclear F-actin, determined by Pearson's correlation coefficient (One-way ANOVA with Tukey's post hoc test; ****: $p < 0.0001$). (C) Nuclear F-actin levels in primary N-WASP KO keratinocytes transfected with the indicated constructs. Values were normalized by dividing transfected cells (TF) by untransfected cells (UTF). Each dot indicates an individual experiment with more than 30 cells analyzed (n= 7, 8, 6, 7, 8, 7, 6; one-way ANOVA with Tukey's post hoc test; not significant (ns): $p > 0.05$; ***: $p < 0.001$).

Figure S3

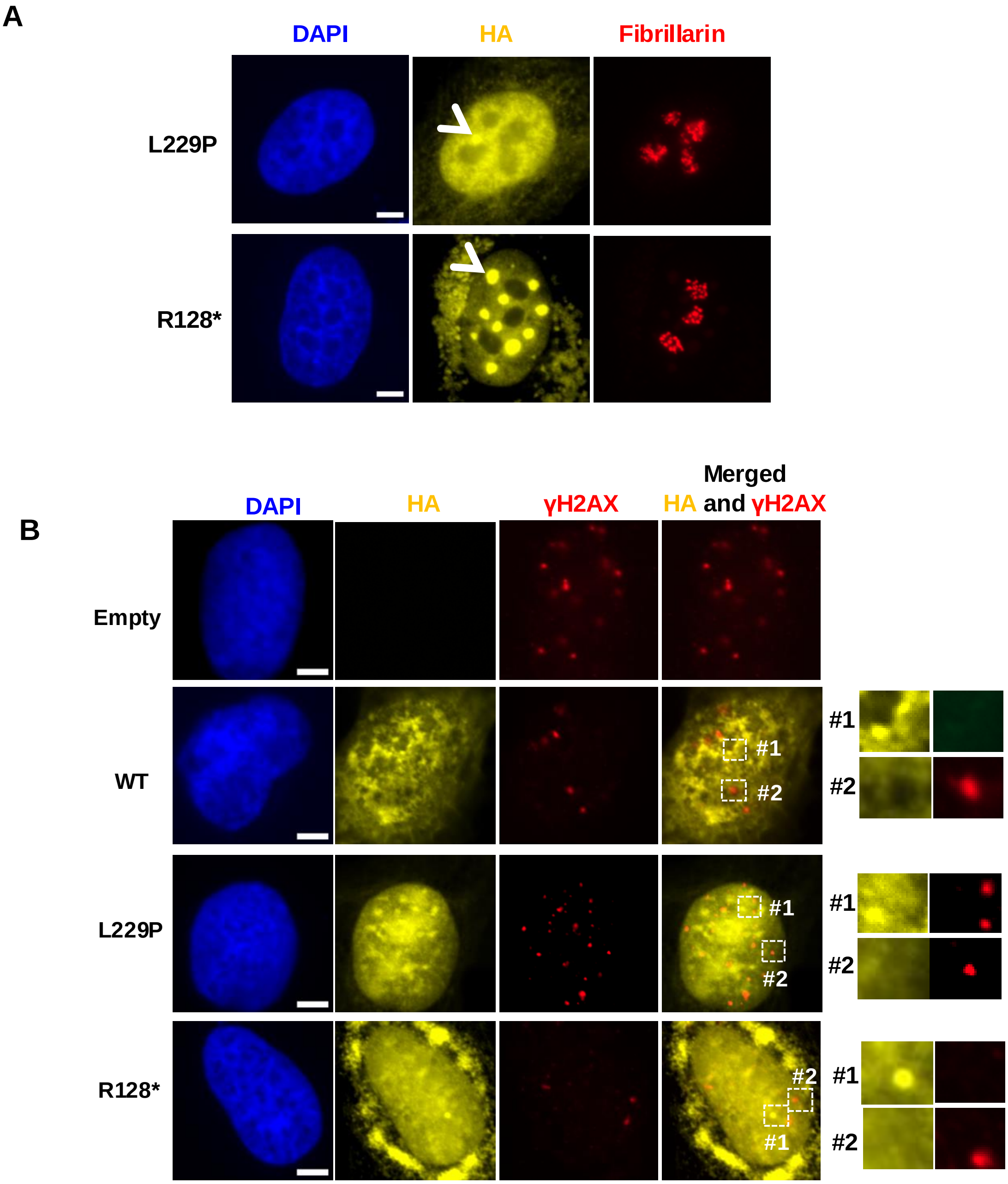
A
DAPI
HA
Fibrillarin
L229P
R128*
B
DAPI
HA
γH2AX
Merged
HA and γH2AX
Empty
WT
#1
#2
L229P
#1
#2
R128*
#1
#2

Figure S3: Nuclear N-WASP nodules do not colocalize with nucleoli or spontaneous γH2AX foci.

(A) Confocal microscopy of U2OS cells transfected with indicated N-WASP constructs and stained for DNA (DAPI), transfected N-WASP (HA), and the nucleoli marker fibrillarin. Nuclear nodules of N-WASP did not co-localize with nucleoli (arrowhead indicates N-WASP nodule; scale bar 5 μm). (B) Confocal microscopy of U2OS cells transfected with indicated N-WASP constructs and stained for DNA (DAPI), transfected N-WASP (HA), and γH2Ax which marks DNA double-strand DNA breaks (DSBs). Nuclear nodules of N-WASP (HA, yellow) do not co-localize with spontaneous DSBs (Scale bar 5 μm). Zoom-in images of nodules are shown near the corresponding images of whole nuclei. More than 15 cells were analyzed per experiment (n: 2).

Figure S4

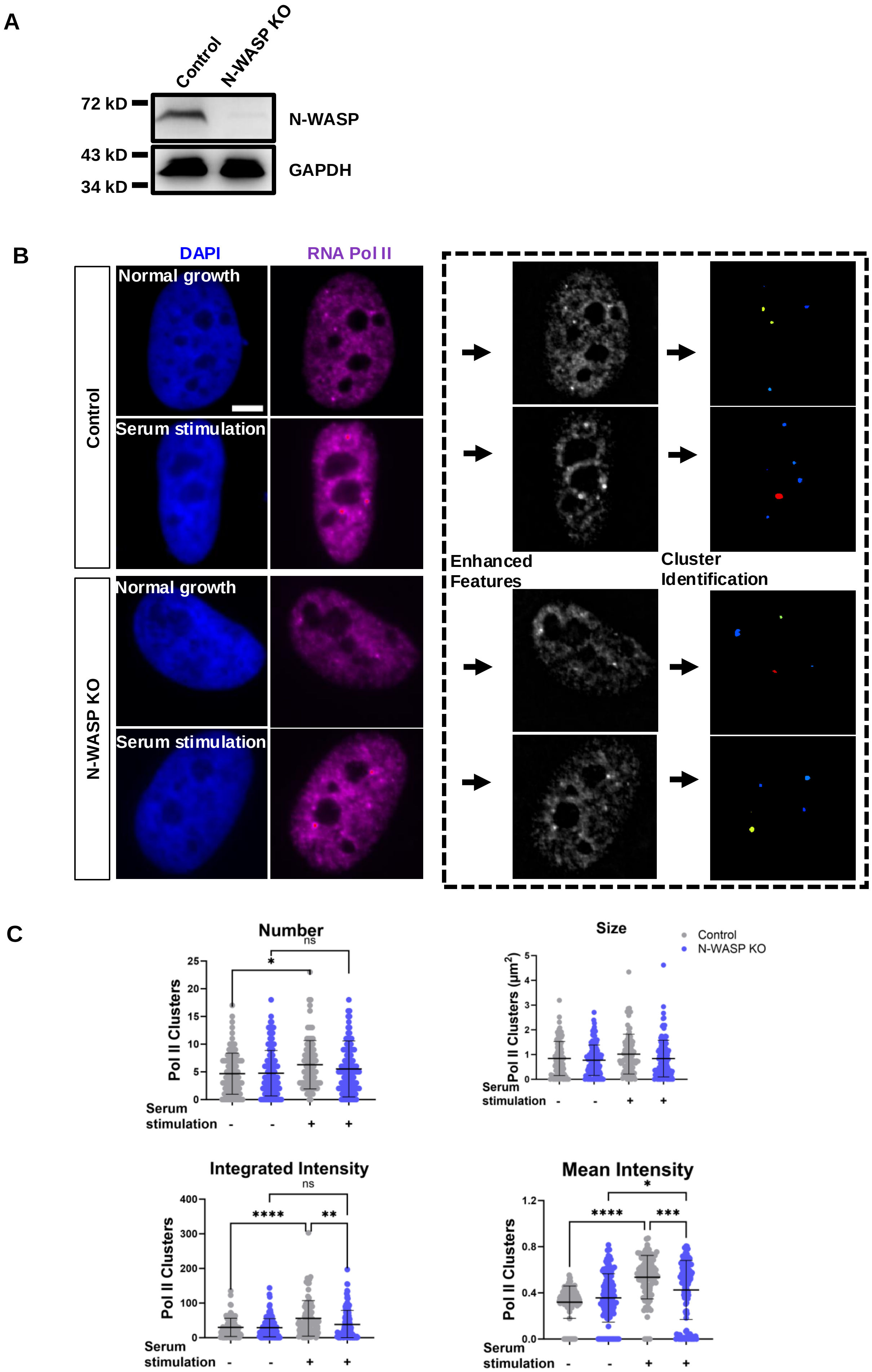
A
Control
N-WASP KO
72 kD
43 kD
34 kD
N-WASP
GAPDH
B
DAPI
RNA Pol II
Control
N-WASP KO
Normal growth
Serum stimulation
Normal growth
Serum stimulation
Enhanced Features
Cluster Identification
C
Number
ns
*
Pol II Clusters
Serum stimulation
- - + +
Size
Control
N-WASP KO
Pol II Clusters (μm²)
Serum stimulation
- - + +
Integrated Intensity
ns
****
**
Pol II Clusters
Serum stimulation
- - + +
Mean Intensity
*
****
***
Pol II Clusters
Serum stimulation
- - + +

Figure S4: Partial requirement of endogenous N-WASP in serum-induced enhancement of RNA Pol II clusters.
(A) Western blot for N-WASP showing efficient loss of N-WASP protein in N-WASP KO U2OS cells. GAPDH was used as loading control. (B) Representative microscopic images displaying RNA Pol II clusters in both control and N-WASP KO U2OS cells, under normal growth and serum stimulation conditions (Scale bar: 5 μm). Images of identified RNA Pol II clusters using CellProfiler are also presented. (C) Quantitative analysis of RNA Pol II cluster characteristics per nucleus (number, size, integrated intensity and mean intensity) in control and N-WASP KO U2OS cells under normal growth (cells analyzed: 99;97) and serum stimulation condition (cells analyzed: 145; 125). Data are pooled from three independent experiments and each dot represents a single nucleus. (One-way ANOVA with Tukey's post hoc test; (not significant (ns): $p > 0.05$; *: $p < 0.05$; **: $p < 0.01$; ***: $p < 0.001$; ****: $p < 0.0001$).

Figure S5

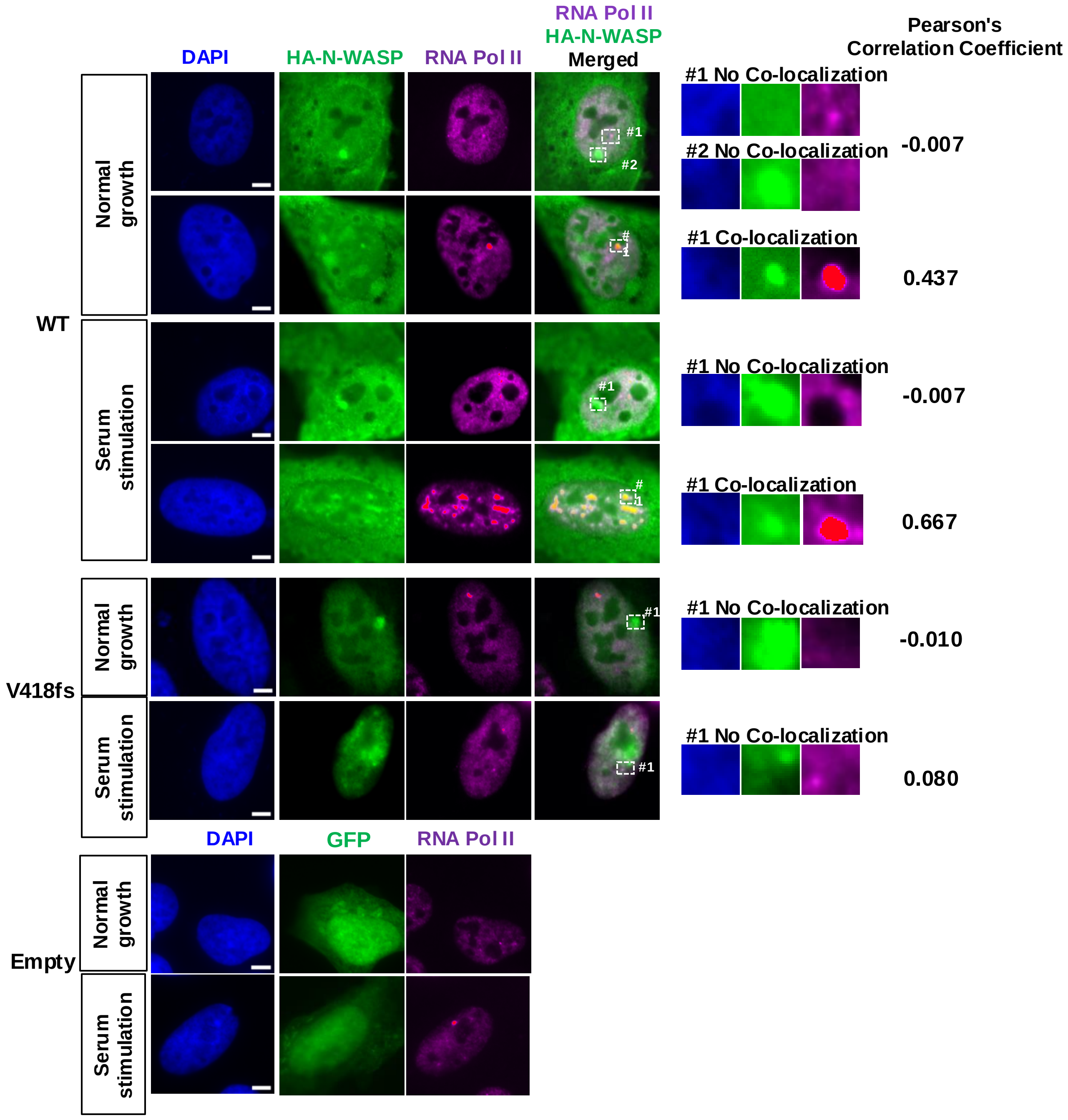

DAPI
HA-N-WASP
RNA Pol II
RNA Pol II
HA-N-WASP
Merged
Pearson's
Correlation Coefficient
WT
Normal growth
Serum stimulation
#1
#2
#1 No Co-localization
#2 No Co-localization
-0.007
#1 Co-localization
0.437
#1 No Co-localization
-0.007
#1 Co-localization
0.667
V418fs
Normal growth
Serum stimulation
#1 No Co-localization
-0.010
#1 No Co-localization
0.080
DAPI
GFP
RNA Pol II
Empty
Normal growth
Serum stimulation

Figure S5. Co-localization of N-WASP nodules with RNA Pol II shows a widespread pattern both in normal growth and serum stimulation.
Representative widefield images of U2OS cells transfected with indicated N-WASP constructs and stained for DNA (DAPI), N-WASP (HA) and RNA Pol II. A white box with a dashed line indicates an RNA Pol II nodule which is zoomed in on the right. RNA Pol II clusters co-localization with N-WASP nodules shows high cell-to cell variation. Colocalization occurred in the presence and absence of serum stimulation (Scale bar: 5 μm). Co-localization was quantified for nuclei of these cells by the by Pearson's correlation coefficient.

Figure S6

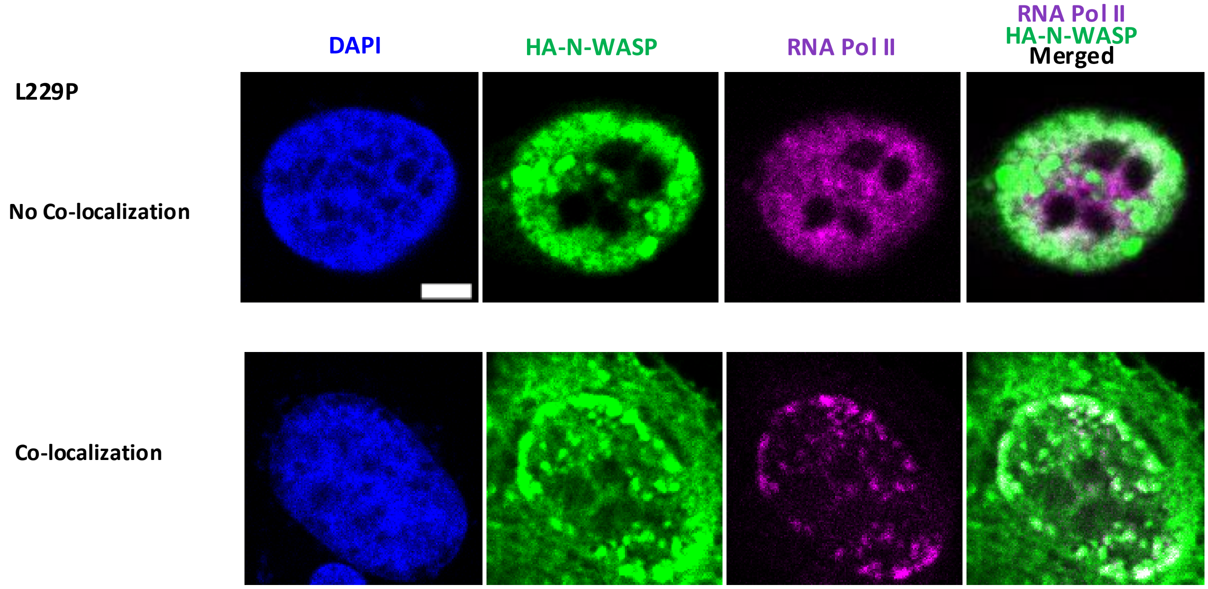
DAPI
HA-N-WASP
RNA Pol II
RNA Pol II
HA-N-WASP
Merged
L229P
No Co-localization
Co-localization

Figure S6. Co-localization of N-WASP L229 nodules with RNA Pol II shows a widespread pattern in serum stimulation.
Representative confocal images of U2OS cells transfected with L229P N-WASP, stiulated with serum, and stained for DNA (DAPI), N-WASP (HA) and RNA Pol II. RNA Pol II clusters co-localization with N-WASP nodules shows high cell-to cell variation. (Scale bar: 5 μm).

Table S1

Antigen	Producer	Cat. Nr.	Dilution
HA-tag	Cell Signaling Technology	C29F4	1:1 000
N-WASP	Cell Signaling Technology	30D10	1:1 000
Cortactin	Cell Signaling Technology	H222	1:1 000
WIP	Santa Cruz Biotechnology	sc-271113	1:1 000
GAPDH	Sigma-Aldrich	G9545	1:10 000
GAPDH	Santa Cruz Biotechnology	sc-32233	1: 1 000
(HRP)-coupled horse anti- mouse IgG	Vector Laboratories	PI-2000	1: 10 000
(HRP)-coupled goat anti-rabbit IgG	Vector Laboratories	PI-1000	1: 10 000

Table S2

Antigen	Producer	Cat. Nr.	Dilution
Mouse WIP	Santa Cruz Biotechnology	sc-271113	1:50
Mouse Fibrillarin	Abcam	ab4566	1:500
Mouse Lamin A/C	Santa Cruz Biotechnology	sc-376248	1:500
Mouse γH2AX	Thermo Fisher Scientific	MA1-2022	1:1 000
Mouse HA tag (overexpressed N-WASP)	Cell Signaling Technology	6E2	1:200
Rabbit HA tag (overexpressed N-WASP)	Cell Signaling Technology	C29F4	1:1 000
Rabbit Cortactin	Cell Signaling Technology	H222	1:50
Rabbit RNA Polymerase II	Abcam	ab193467	1:250
Rabbit N-WASP (endogenous)	Thermo Fisher Scientific	PA5-52198	1:1 000
Anti-GFP Alexa Fluor 488	Thermo Fisher Scientific	A-21311	1:200
Alexa Fluor 546-coupled goat anti mouse IgG	Thermo Fisher Scientific	A-11030	1: 1 000
Alexa Fluor 647-coupled goat anti mouse IgG	Thermo Fisher Scientific	A-21235	1: 1 000
Alexa Fluor 568-coupled goat anti rabbit IgG	Thermo Fisher Scientific	A-11011	1: 1 000
Alexa Fluor 633-coupled goat anti rabbit IgG	Thermo Fisher Scientific	PA5-52198	1: 1 000
Alexa Fluor 488-coupled goat anti rabbit IgG (for N-WASP)	Thermo Fisher Scientific	A-11008	1: 1 000

Table S3

Confocal microscopy settings for using the Plan-Apochromat, 40x / 1.3 oil objective of the Zeiss LSM 800

Module	DAPI	GFP (Alexa488)	HA-N-WASP (Alexa568)	Phalloidin647
Pinhole	1.48 AU / 42 µm	1.35 AU / 42 µm	1.14 AU / 42 µm	1.35 AU / 42 µm
Laser Wavelength	405 nm: 0.7 %	488 nm: 0.2 %	561 nm: 0.3 %	640 nm: 0.3 %
Scan Mode	Frame	Frame	Frame	Frame
Scan Zoom	1.0	1.0	1.0	1.0
Scan Direction	undirectional	undirectional	undirectional	undirectional
Channel Name	DAPI-T3	EGFP-T2	AF568-T1	AF647-T2
Dye Name	DAPI	EGFP	AF568	AF647
Excitation Wavelength	353	488	577	653
Emission Wavelength	465	509	603	668
Detection Wavelength	400-495	410-546	560-640	650-700
Imaging Device	GaAsP-Pmt1	GaAsP-Pmt1	Airyscan	GaAsP-Pmt2
Detector Type	GaAsP	GaAsP	Airyscan	GaAsP
